# Surface Immobilization of Anti-VEGF Peptide on SPIONs for Antiangiogenic and Targeted Delivery of Paclitaxel in Non-Small-Cell Lung Carcinoma

**DOI:** 10.1021/acsabm.3c00224

**Published:** 2023-06-29

**Authors:** Lindokuhle M. Ngema, Samson A. Adeyemi, Thashree Marimuthu, Philemon N. Ubanako, Wilfred Ngwa, Yahya E. Choonara

**Affiliations:** † Wits Advanced Drug Delivery Platform Research Unit, Department of Pharmacy and Pharmacology, School of Therapeutic Sciences, Faculty of Health Sciences, University of the Witwatersrand, 7 York Road, Parktown, Johannesburg 2193, South Africa; ‡ Sidney Kimmel Comprehensive Cancer Center, School of Medicine, Johns Hopkins University, Baltimore, Maryland 21218, United States

**Keywords:** angiogenesis, vascular endothelial growth factor targeting, HRH peptide, SPIONs, paclitaxel, non-small-cell lung carcinoma

## Abstract

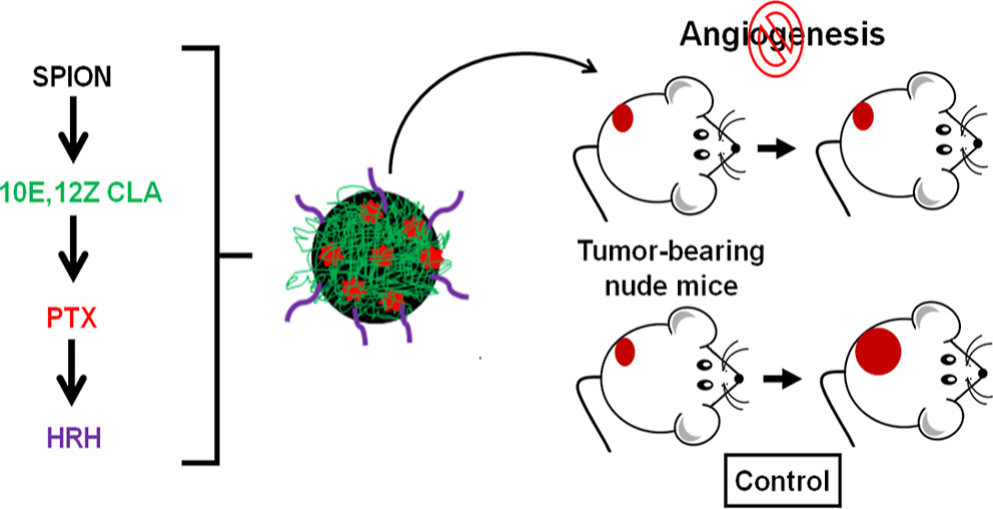

A design has been established for the surface decoration of superparamagnetic iron oxide nanoparticles (SPIONs) with anti-vascular endothelial growth factor peptide, HRH, to formulate a targeted paclitaxel (PTX) delivery nanosystem with notable tumor targetability and antiangiogenic activity. The design methodology included (i) tandem surface functionalization *via* coupling reactions, (ii) pertinent physicochemical characterization, (iii) *in vitro* assessment of drug release, anti-proliferative activity, and quantification of vascular endothelial growth factor A (VEGF-A) levels, and (iv) *in vivo* testing using a lung tumor xenograft mouse model. Formulated CLA-coated PTX-SPIONs@HRH depicted a size and surface charge of 108.5 ± 3.5 nm and −30.4 ± 2.3 mV, respectively, and a quasi-spherical shape relative to pristine SPIONs. Fourier transform infrared (FTIR) analysis and estimation of free carboxylic groups supported the preparation of the CLA-coated PTX-SPIONs@HRH. CLA-coated PTX-SPIONs@HRH exhibited high PTX loading efficiency (98.5%) and sustained release *in vitro*, with a marked dose dependent anti-proliferative activity in A549 lung adenocarcinoma cells, complimented by an enhanced cellular uptake. CLA-coated PTX-SPIONs@HRH significantly reduced secretion levels of VEGF-A in human dermal microvascular endothelial cells from 46.9 to 35.6 pg/mL compared to untreated control. A 76.6% tumor regression was recorded in a lung tumor xenograft mouse model following intervention with CLA-coated PTX-SPIONs@HRH, demonstrating tumor targetability and angiogenesis inhibition. CLA-coated PTX-SPIONs@HRH enhanced the half-life of PTX by almost 2-folds and demonstrated a prolonged PTX plasma circulation time from a subcutaneous injection (SC). Thus, it is suggested that CLA-coated PTX-SPIONs@HRH could provide a potential effective treatment modality for non-small-cell lung carcinoma as a nanomedicine.

## Introduction

Non-small-cell lung carcinoma (NSCLC) continues to drive the global prevalence of lung cancer, as it constitutes ∼85% of all reported lung cancer cases.^1^ To date, the treatment of NSCLC remains a great challenge. Over the years, conventional treatment modalities for NSCLC, including combination chemotherapy, have been shown to be riddled with exigent shortcomings such as non-specificity, high dosages, and intolerable side effects, which have culminated in their therapeutic plateau.^2,3^ Particularly, paclitaxel (PTX), as a first-line chemotherapeutic drug prescribed to NSCLC patients is challenged with poor solubility, short half-life, and undesired binding to tissue-proteins.^3^ Consequently, effective delivery strategies are needed to address these shortcomings. The approach of targeted nanomedicine presents a promising avenue, which allows for specific targeting of key tumor biomarkers and the delivery of drugs directly to target tumors.^4^

Angiogenesis is one of the crucial pathways in tumor growth, responsible for the provision of oxygen and key nutrients, promoting tumor vascularization and metastasis.^5,6^ Therefore, the inhibition of angiogenesis through targeting of its biomarkers can potentially halt tumor proliferation and metastasis in NSCLC.^7^ Vascular endothelial growth factor (VEGF), encompassing VEGF A–D, is a vital biomarker and regulator of angiogenesis, which binds vascular endothelial growth receptors (VEGFR 1–3) to initiate formation of new blood vessels.^5,8^ As such, antiangiogenic therapy has manifested as a potent therapeutic intervention in the management of NSCLC through either blocking of VEGF/VEGFR binding or suppressing VEGFR-mediated downstream signaling through tyrosine kinase inhibitors.^9^

The peptide HRH (HRHTKQRHTALH) was first reported by Zhang *et al.*, in 2017, and demonstrated high affinity for VEFGRs, *in vitro* and *in vivo*.^10^ The novel peptide was identified using VEGFR-Fc fusion protein from the Ph.D-12 phage library screening and assessed for antiangiogenic activity on human endothelial cells and rodent models. HRH was found to be a mimotope that mimicked the binding sites of VEGF on VEGFRs, thus competitively inhibiting the binding of VEGF A, B, and C to VEGFR 1 and 2 and subsequently halting angiogenesis.^10^ Later, Chen *et al.*, 2021, explored a chemically functionalized derivative of HRH to enable fabrication of nanofibers. The nanofibers demonstrated excellent *in vitro* and *in vivo* antiangiogenic activity relative to HRH administered under the same conditions^11^ Ideally, the incorporation of HRH, as a homing peptide, onto a drug delivery nanosystem could yield an efficacious targeted nanomedicine, with the ability to target tumors (highly expressing VEGFRs) to block angiogenesis while releasing the anticancer drug, thus halting tumor growth and metastasis.

Previously, we have reported on the formulation of novel *trans*-10,*cis*-12 conjugated linoleic acid (CLA)-coated superparamagnetic iron oxide nanoparticles (SPIONs) loaded with paclitaxel (PTX), designated CLA-coated PTX-SPIONs, as a potential nanomedicine with enhanced anti-proliferative activity for NSCLC intervention.^12^ Essentially, SPIONs are superior drug vehicles, presenting with high drug-loading capacity, compatibility, and smaller size (suitable for lung tumor penetration).^13,14^ Interestingly, *trans*-10,*cis*-12 CLA (10E, 12Z CLA) is a natural fatty acid exhibiting anticancer properties, shown to disrupt lipid uptake and metabolism in cancerous cells, resulting in suppression of cell growth.^15^

Moreover, 10E, 12Z CLA is an excellent coating agent, allowing maximal partitioning of PTX, a hydrophobic anticancer drug, owing to its poor aqueous solubility.^12,16^ SPIONs coated with CLA have demonstrated enhanced antitumor activity against mouse breast cancer cells (4T1).^17^ Meanwhile, peptides generally exhibit high selectivity and good efficacy for biomedical application.^11,18^ This study reports on a holistic fabrication approach in achieving a targeted nanosystem that is safe by design, through the formulation of HRH-functionalized CLA-coated PTX-SPIONs (CLA-coated PTX-SPIONs@HRH) and its *in vitro* and *in vivo* evaluation. Subcutaneous injection of CLA-coated PTX-SPIONs@HRH in a lung tumor xenograft mouse model halted angiogenesis and restricted tumor growth.

## Results and Discussion

### Fabrication of CLA-Coated PTX-SPIONs@HRH

The use of surface-engineered SPIONs as drug delivery vehicles is currently a promising modality for targeted therapy and management of NSCLC.^19^ Essentially, surface modification is key in the application of SPIONs as anticancer drug vehicles, as it allows customization of SPIONs according to tumor specificity.^13^ Primarily, SPIONs synthesized *via* a co-precipitation method have enormous surface OH^–^ groups that can be manipulated to allow desired surface modifications, *via* chemisorption chemistry.^12,20^ Accordingly, the active carboxyl group and inherent crosslinking activity of 10E, 12Z CLA was exploited for chemisorption onto the SPIONs for surface coating, while the hydrophobicity of the isomer allowed adsorption of hydrophobic PTX. Fundamentally, the understanding of the coating material as well as the loading technique is very critical in drug loading in order to maximize drug entrapment, ensure that the functionality of the drug is not compromised, and achieve sustained deposition of the drug on the site.^21^

**Figure 1:**
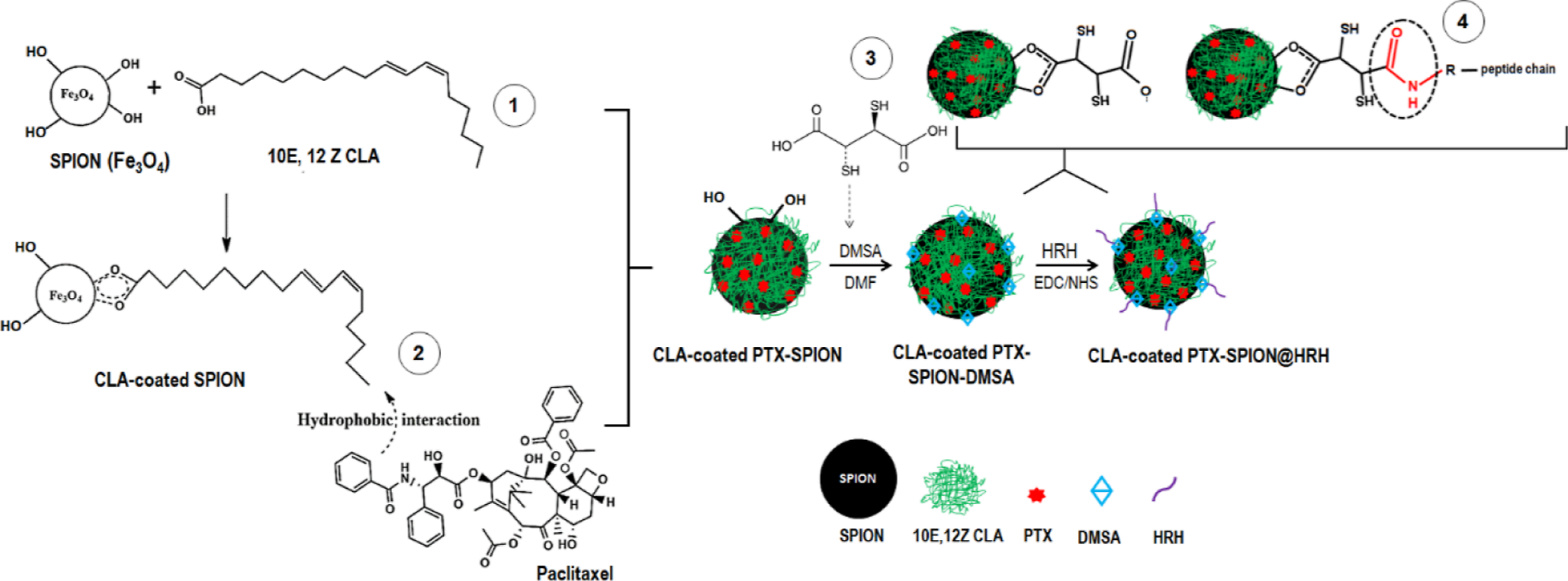
Schematic illustration of the fabrication methodology of CLA-coated PTX-SPIONs@HRH (not drawn to actual scale). The coating of SPIONs with 10E, 12Z is through chemisorption *via* −COOH of CLA and −OH on the SPION surface. PTX loading onto CLA-coated SPIONs is *via* a favored hydrophobic–hydrophobic interaction between CLA and PTX. The physical functionalization of the SPION surface with DMSA was carried out in DMF to favor the hydrogen bonding of the −COOH groups in DMSA and available adsorbed −OH groups on the SPION surface.^25,26^ Subsequently, the HRH peptide has a reactive −NH_2_ terminal, which enabled conjugation to surface bound −COO^–^ groups on SPIONs after EDC/NHS activation.

A CLA-coated PTX-SPIONs@HRH nanosystem was successfully fabricated from black powdered, purely magnetite SPIONs (Fe_3_O_4_) synthesized from iron chloride salts, coated with a hydrophobic 10E, 12Z CLA isomer, partitioned with hydrophobic PTX, and functionalized with HRH peptide *via* a DMSA-aided EDC/NHS chemistry conjugation (Figure 1). The content of 10E, 12Z CLA coated onto SPIONs was found to be 10.3%, which resulted in 98.5% PTX adsorption efficiency (% AE) and 9.8% drug loading capacity (% DLC; amount of PTX per unit mass of nanoformulation). The % AE and % DLC based on the use of 10 mg of PTX for drug loading (98.5 and 9.8%, respectively) supported the use of 10E, 12Z CLA as a suitable partitioning agent for PTX, and arguably for other hydrophobic anticancer drugs, and supports the concept that SPIONs offer high drug loading capacity.^2,22^ Moreover, the latter can be confirmed from the present study, considering that 10% (*w*/*w*) PTX was used in the loading process and actually 9.8% of that could be successfully loaded onto CLA-coated SPIONs.

Carboxylic acid functionalization by DMSA was a crucial step in the fabrication of CLA-coated PTX-SPIONs@HRH, which required the use of a polar aprotic solvent DMF to enable DMSA grafting (*via* hydrogen bonding) onto SPIONs. Ideally, a polar aprotic solvent allows the coupling reaction to occur without it partaking in hydrogen bonding with the nucleophile.^23^ The number of free −COOH was ascertained to be 63.7 per gram of CLA-coated PTX-SPIONs which could be expected from 1.6 M of DMSA. Dilnawaz *et al.*, 2010, previously reported acid numbers from 8 per g of glycerol monooleate (GMO)-coated Fe_3_O_4_ when 0.2 M DMSA was used, up to 130 acid number with increase in DMSA concentration, however, noted that once binding saturation is reached, even the increase in DMSA concentration does not significantly alter acid numbers.^20^ Another study by Garkhal *et al.*, 2007, reported acid number of 44.4 for conjugation of P-15 peptide on poly(l-lactide-*co*-ε-caprolactone) modified microspheres.^24^ The molar ratio of EDC/NHS to free −COOH was fixed at 1:0.5 to allow for adequate activation of the −COOH groups and conjugation of HRH peptide. The methodology exhibited high efficacy with a recorded % HRH conjugation of 66.6%. The successful conjugation of HRH peptide was also evident from the qualitative Fourier transform infrared (FTIR) analysis (Figure 3e).

### Particle Size and Overall Morphological Analysis of CLA-Coated PTX-SPIONs@HRH

**Figure 2:**
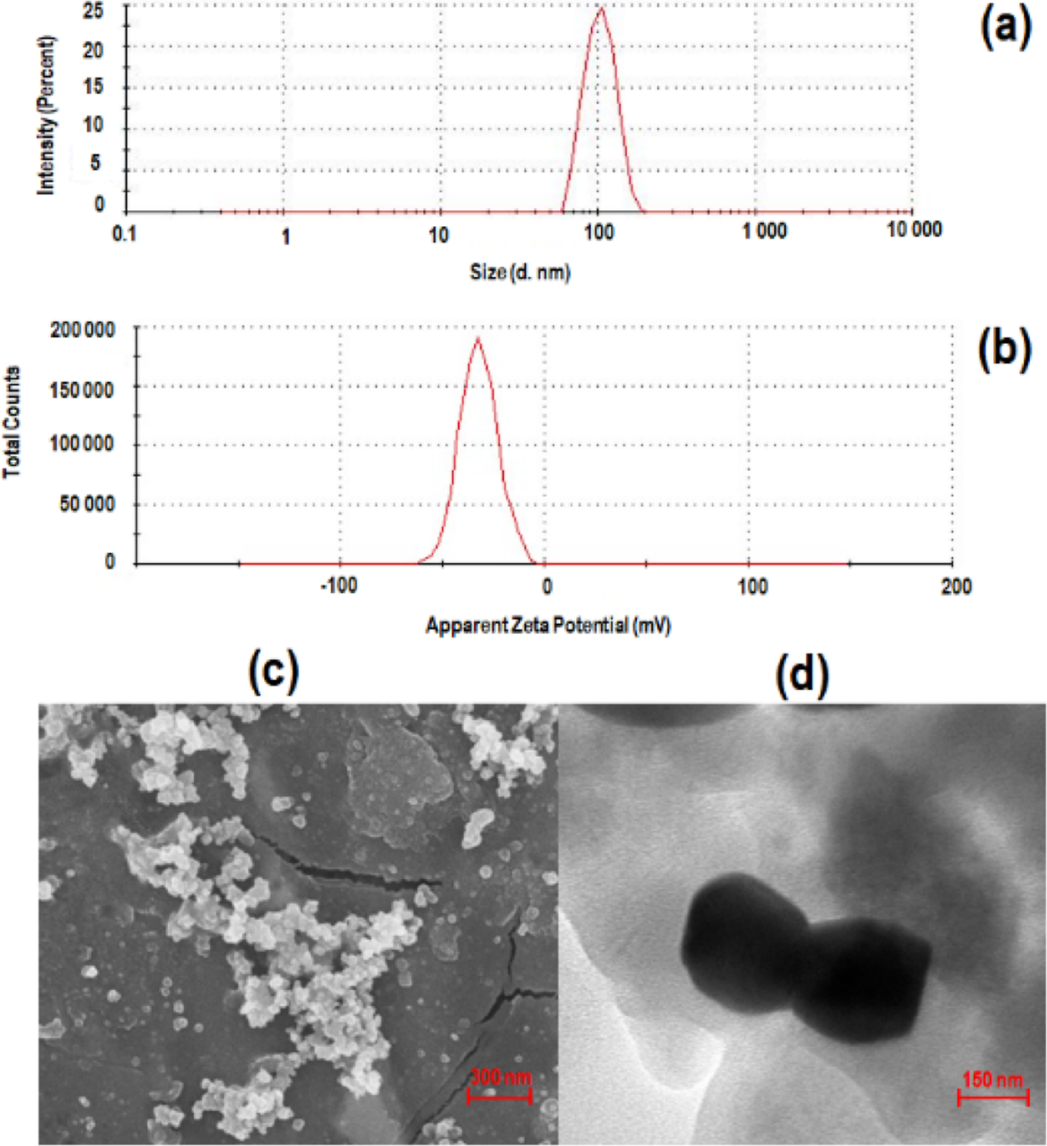
Graphical representation of (a) mean hydrodynamic size (108.5 ± 3.5 nm) and (b) zeta potential (−30.4 ± 2.3 mV) of CLA-coated PTX-SPIONs@HRH, with corresponding morphological depiction by (c) SEM viewed at the 300 nm scale (magnification = 81.75 KX) and (d) TEM viewed at the 150 nm scale (magnification = 73,000 X). Captured micrographs show quasi-spherical shaped nanoparticles in the nm size range, and within the specified average size, as shown by the scale bars.

The mean particle size and surface charge of formulated CLA-coated PTX-SPIONs@HRH was found to be 108.5 ± 3.5 nm and −30.4 ± 2.3 mV, respectively (Figure 2a,b), with a polydispersity index (PDI) of 0.2 ± 0.01. The zeta potential and PDI showed that CLA-coated PTX-SPIONs@HRH exhibited colloidal stability with reduced aggregation in aqueous media, which fits the criteria for biomedical application.^27^ Interestingly, the particle size obtained is relatively smaller compared to other reported ligand-functionalized targeted nanosystems for NSCLC, such as tumor homing peptide *LYP*-1 functionalized liposome nanoparticles (*tLYP*-1-*PEG*-*NPs*; 188 nm) by Jin *et al.*, 2018,^28^ and an antibody modified targeted nanostructured carrier (*Flk*-1-*DSPE*-*PEG*-*NH*_2_-*NLC*; 168 nm) by Liu *et al.*, 2011,^29^ among others. Essentially, the smaller particle size is ideal for lung tumor penetration.^30^ The overall morphology, as examined by using a scanning electron microscope (SEM) and transmission electron microscope (TEM) (Figure 2c,d), revealed a quasi-spherical shape for CLA-coated PTX-SPIONs@HRH with a slightly rugged surface and a nm size range. The quasi-spherical shape could be attributed to surface modification by DMSA, with short chains of DMSA around the spherical iron oxide imparting the quasi-spherical conformation, as mostly reported for DMSA-modified iron oxide nanoparticles.^31^

### Analysis of Chemical Composition and Functional Transformations of CLA-Coated PTX-SPIONs@HRH

**Figure 3:**
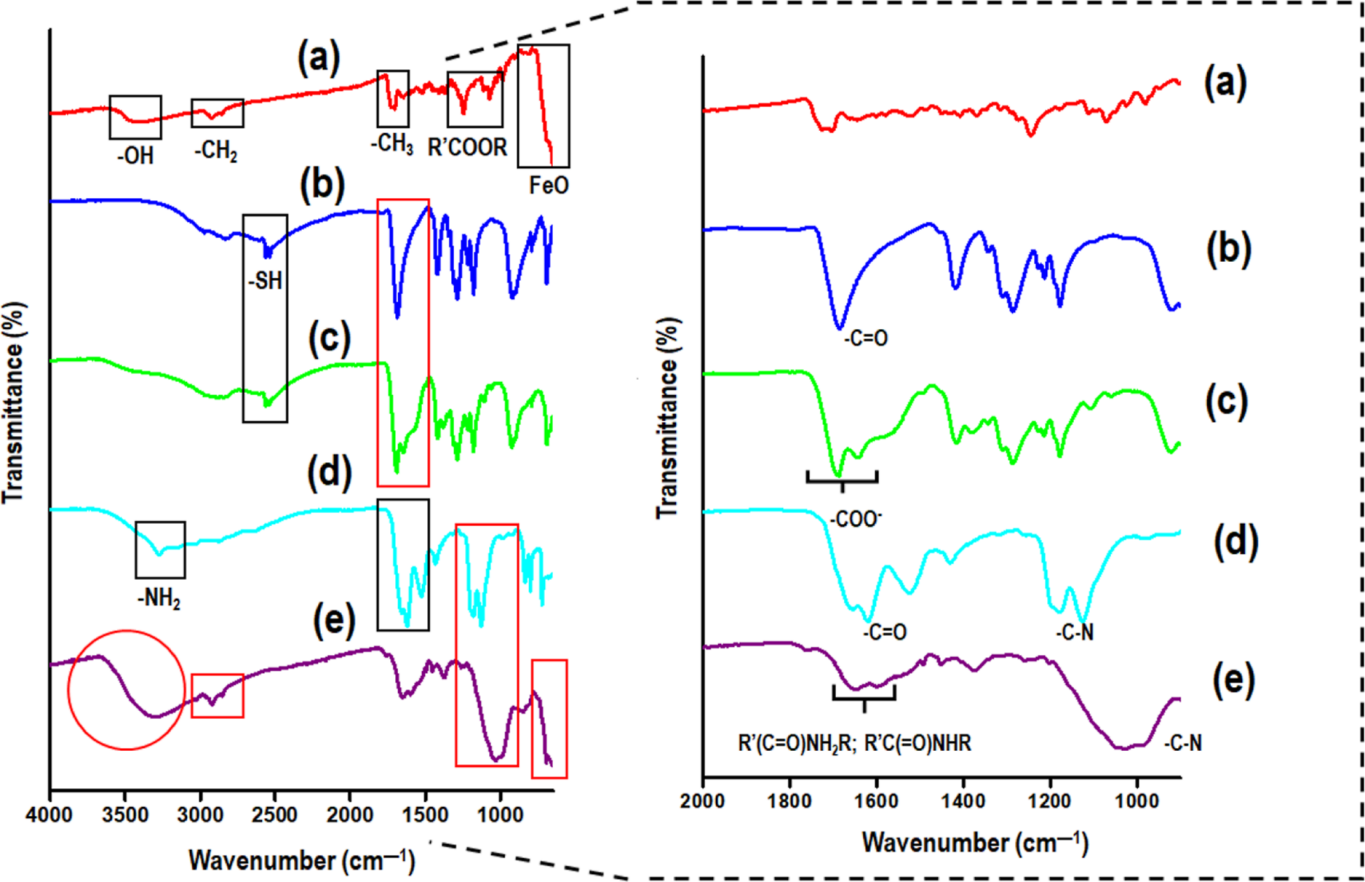
Functional group mapping by FTIR spectroscopy showing identified characteristic functionality of (a) CLA-coated PTX-SPIONs, (b) DMSA, (c) CLA-coated PTX-SPIONs-DMSA, (d) HRH, and (e) CLA-coated PTX-SPIONs@HRH. On the right is the expanded image at 2000–1000 cm^–1^.

The chemical composition of CLA-coated PTX-SPIONs, DMSA, and HRH, as well as functional transformations toward the formation of CLA-coated PTX-SPIONs@HRH were mapped by FTIR spectroscopy. Essentially, FTIR spectroscopy has proven to be a robust technique for immediate identification of functional groups in compounds and characterization of bond formations.^26^ The FTIR spectra obtained in the present study are shown in Figure 3a–e. The spectrum for CLA-coated PTX-SPIONs (Figure 3a) exhibited characteristic PTX peaks at 3479 and 1244 cm^–1^ (belonging to −OH and ester bonds, respectively), characteristic 10E, 12Z CLA peaks around 2922–2852 cm^–1^ belonging to ν_sym_/ν_asym_ −CH_2_ stretch vibrations, and 1408 cm^–1^ belonging to a CH_3_ bending, as well as an iron oxide (Fe–O) peak at around 580 cm^–1^.^17,32^ Characteristic peaks of DMSA (Figure 3b) were observed at 2561 and 1685 cm^–1^ assigned to the thiol (−SH) and C=O of the carboxyl groups, respectively.^33^

In a spectrum of CLA-coated PTX-SPIONs-DMSA (Figure 3c), a split in the C=O peak of DMSA to 1685 and 1647 cm^–1^ was observed, presumably belonging to the stretching vibrations of the carboxylate (COO^–^) ions due to DMSA linkage onto the SPION surface *via* the carboxyl group (Figure 1).^33,34^ A spectrum of pure HRH peptide (Figure 3d) exhibited characteristic peaks at around 3276, 1654–1619, and 1261–1255 cm^–1^ assigned to the terminus primary amine (−NH_2_), C=O stretch, and peptide bond (C–N) stretch, respectively.^35^ Meanwhile, CLA-coated PTX-SPIONs@HRH (Figure 3e) exhibited major characteristic peaks of all starting materials (red rectangle), with overlapping bands at ∼1652 and 1570 cm^–1^ assigned to amide I and II, respectively.^36^ Particularly, the amide II [R’C(=O)NHR] band could be associated with HRH conjugation *via* −NH_2_ onto activated COO^–^ offered by DMSA (Figure 1). Moreover, a new broad band at 3302 cm^–1^ was identified (red circle), corresponding to N–H stretching resulting from HRH conjugation, meanwhile a shift in C–N stretch (∼1028 cm^–1^) could be associated with increasing peptide bonds, owing the new amide II band formation to a new bond formation. This qualitatively confirmed the conjugation of HRH peptide onto the nanosystem, and together with the plausible % HRH conjugation obtained supports the effectiveness of the method for decoration of CLA-coated PTX-SPIONs with HRH peptide.

### *In Vitro* PTX Release Kinetics from CLA-Coated PTX-SPIONs@HRH

**Figure 4:**
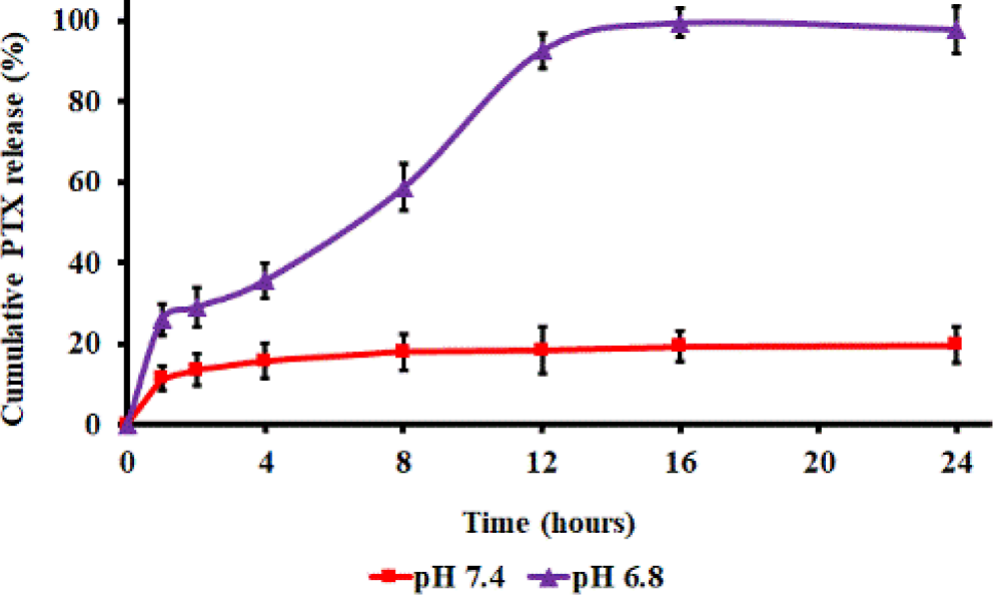
*In vitro* cumulative release of PTX at pH 6.8 and pH 7.4 over 24 h. Data presented as mean ± SD of three sample analyses (*n* = 3) per time point.

**Figure 5:**
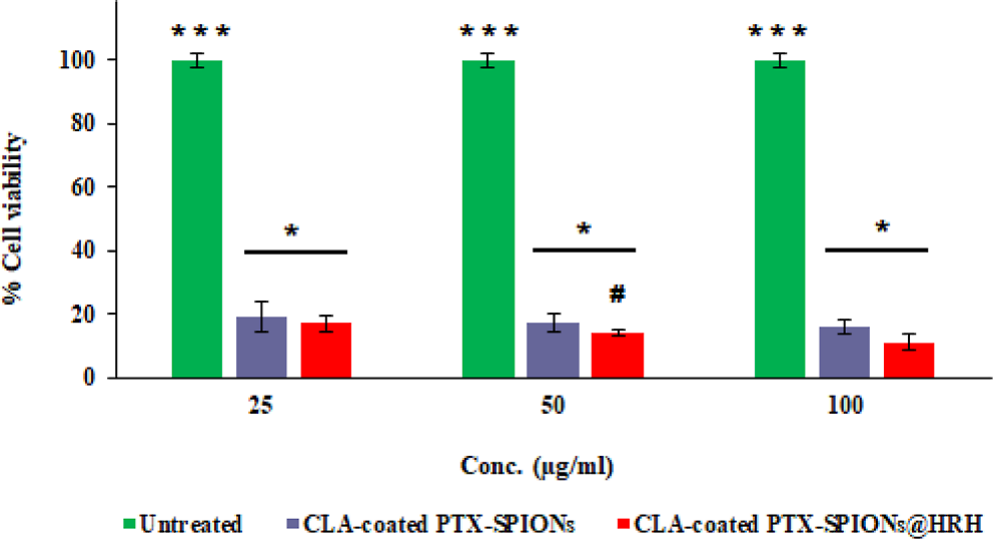
Cell viability (%) profile of A549 cells following treatment with varying concentrations of CLA-coated PTX-SPIONs and CLA-coated PTX-SPIONs over 72 h. The treatment concentrations were based on the concentrations that could yield distinguishable outcome between the HRH-functionalized and non-functionalized nanosystem, to discern the effect of the peptide. Data computed as mean ± SD, *n* = 3 (*** depicts *p* < 0.001, compared to treatment groups at each concentration, * depicts *p* < 0.05 among all concentrations, and # depicts *p* < 0.05, against CLA-coated PTX-SPIONs at a specified concentration).

The release profile of PTX from CLA-coated PTX-SPIONs@HRH over 24 h is presented in Figure 4. The fabricated nanosystem exhibited a sustained release of PTX at pH 6.8, with maximal cumulative release of 99.4% recorded at 16 h. The sustained PTX release at acidic pH is consistent with previous studies which have reported a similar release behavior when PTX is loaded onto fatty acids such as oleic acid and its derivatives.^37^ The high and sustained release of PTX at pH 6.8 could be attributed to the weakening of the hydrophobic–hydrophobic strength between CLA ∼ PTX at relatively acidic pH, thus allowing PTX to detach from CLA ends and is released over time.^12^ Meanwhile, the PTX release was comparatively lower at physiological pH 7.4, with only 19.6% of PTX released in over 24 h. This low cumulative release rate relates to the stability of the hydrophobic CLA-PTX complex at physiological pH, keeping PTX intact over time. The initial release at the first hour was 11.4%, and only 19.6% of PTX was released at 24 h, which is even lower than the release in the first h (25.9%) at pH 6.8. Primarily, the *in vitro* release profile demonstrates that the discharge of PTX from the nanosystem is site-specific. The stimuli-responsiveness of 10E, 12Z CLA to acidic pH 6.8 (mimicking tumor microenvironment) imparts a crucial aspect to the formulated nanosystem, enabling site-specific release of PTX, which subsequently yields robust anti-proliferative action on A549 cells *in vitro* (Figure 5).

### Anti-proliferative Action of CLA-Coated PTX-SPIONs@HRH on A549 Lung Adenocarcinoma Cells

The anti-proliferative merit of CLA-coated PTX-SPIONs@HRH was assessed on A549 lung adenocarcinoma cells, and the treatment response (% cell viability) was recorded and is presented in Figure 5. The anti-proliferative activity was concentration dependent, with a decline in % cell viability as the concentration increased. The maximal therapeutic concentration (100 μg/mL) of CLA-coated PTX-SPIONs@HRH and CLA-coated PTX-SPIONs culminated in % cell viability of 12.8 and 17.1%, respectively. Meanwhile, the cells treated with the minimal concentration (25 μg/mL) of CLA-coated PTX-SPIONs@HRH and CLA-coated PTX-SPIONs had a % cell viability of 17.8 and 19.3%, respectively. This showed that both formulations comparatively suppressed A549 cell proliferation over 72 h; however, CLA-coated PTX-SPIONs@HRH resulted in an enhanced suppression of A549 cells, which is attributed to the effect of HRH peptide on cancer cell proliferation. There is already compelling evidence in literature that HRH peptide suppresses cancer cell proliferation *in vitro* by halting angiogenesis.^10^ Moreover, the results obtained support our previous findings which showed that CLA-coated PTX-SPIONs confer enhanced anti-proliferative activity on A549 cells owing to additional anticancer activity of 10E, 12Z CLA in synergy with PTX.^12^

### Cellular Uptake and Internalization of CLA-Coated PTX-SPIONs@HRH

**Figure 6:**
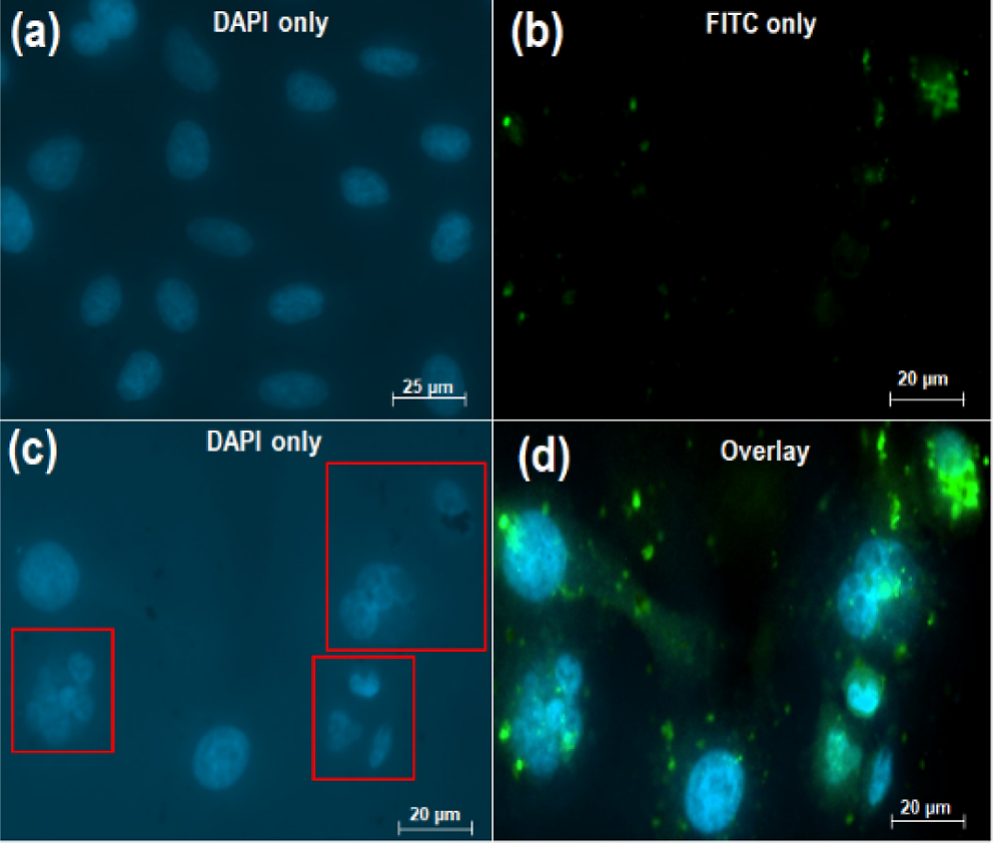
Fluorescent micrographs from a CLSM, showing (a) intact nuclei of untreated control cells, (b) visualization of FITC-labeled CLA-coated PTX-SPIONs@HRH on treated cells, (c) ruptured nuclei of treated cells, and (d) overlay of DAPI and FITC showing the uptake of CLA-coated PTX-SPIONs@HRH by A549 lung adenocarcinoma cells. The ruptured nuclei architecture of treated cells (red boxes) could be distinguished from the normal nuclei architecture of untreated cells.

The surface conjugation of CLA-coated PTX-SPIONs with VEGFR-targeting HRH peptide conferred specificity to the nanosystem for achieving selective uptake by A549 cells. Such cancer cells overexpress VEGFRs, among other angiogenic factors, which propagate cell proliferation and metastasis.^38^ A high uptake of CLA-coated PTX-SPIONs@HRH, sufficient to cause detrimental nuclei damage was observed in model A549 lung adenocarcinoma cells (Figure 6). The DAPI staining (blue fluorescence) was solely employed to visualize the nuclei of untreated cells (Figure 6a) and treated cells (Figure 6c). Meanwhile, FITC (green fluorescence) was used to visualize the nanoformulation (Figure 6b) and track the cellular uptake, as shown on the superimposed (DAPI + FITC) micrograph in Figure 6d. Ideally, a receptor-targeted nanosystem should be able to achieve selective binding to target receptors and robust uptake by target cells.^39^

The proximity of the green fluorescence to the blue fluorescence (Figure 6d) compellingly showed that the nanoformulation was internalized and reached the nuclei of the cells. CLA-coated PTX-SPIONs@HRH could be visualized within the cells, engulfing the nuclei and rupturing the nuclei architecture (red boxes), subsequently resulting in presumed cell death. This revelation compliments and further explains the superlative anti-proliferative activity exhibited by CLA-coated PTX-SPIONs@HRH, as recorded on the MTT assay. Essentially, the high uptake of CLA-coated PTX-SPIONs@HRH observed could be attributed to the preferential binding of HRH onto VEGF receptors on the cellular surface, thus facilitating a receptor-mediated internalization by A549 cells. Moreover, the presence of 10E, 12Z could be implicated in increasing the affinity of A549 cells for CLA-coated PTX-SPIONs@HRH, as cancer cells normally take up essential fatty acids as an energy source.^40^ Similarly, the smaller particle size could have favored the uptake by the cells.^41^

### Quantification of VEGF-A Levels *via* ELISA

**Figure 7:**
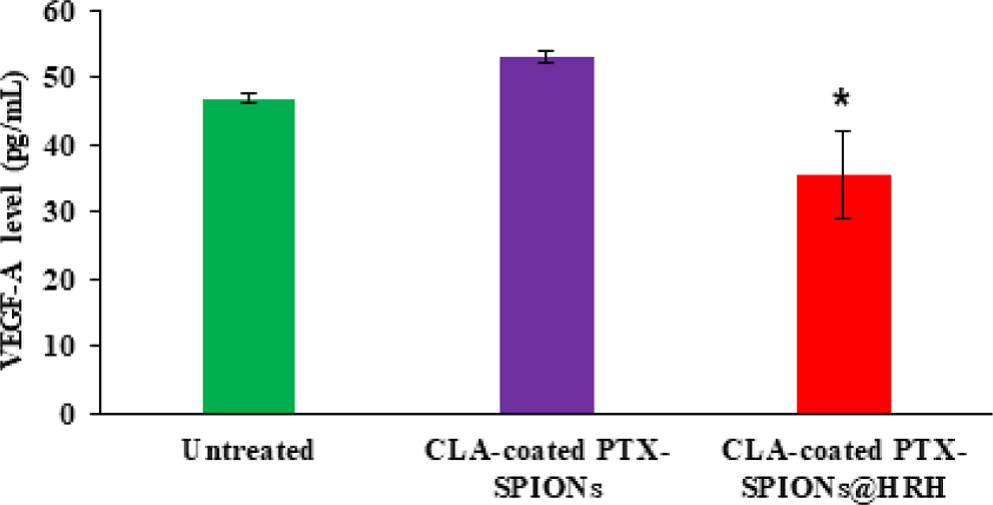
VEGF-A levels from HMEC-1 after 24 h. Data depicted as mean ± SD (* depicts *p* < 0.05, statistically significant compared to untreated and CLA-coated PTX-SPIONs groups). Pristine HRH did not show any statistically significant difference compared to CLA-coated PTX-SPIONs@HRH (data not shown).

VEGF-A predominantly regulates angiogenesis and its levels are perfectly maintained in endothelial cells, such that even a slight disruption may result in lethality.^42^ Herein, the levels of VEGF-A were quantified from endothelial cells (HMEC-1) treated with nanoformulations (with and without HRH peptide) as a measure of antiangiogenic activity through VEGFR blockage. The VEGF-A ELISA concentration plot is presented in Figure 7. The levels of secreted VEGF-A varied among the groups after 24 h, ranging from 35.6 to 53.1 pg/mL. This range could be expected from the endothelial cells as a marker of physiological angiogenesis rather than a pathological angiogenesis (*i.e.*, in cancer and other diseases). It is reported that the over expression of VEGF in cancerous cells could be quantified at much elevated levels of about 310 pg/mL and above.^43^

The level of VEGF-A from untreated cells was 46.9 pg/mL while that from cells treated with CLA-coated PTX-SPIONs and CLA-coated PTX-SPIONs@HRH was 53.1 and 35.6 pg/mL, respectively. A nanoformulation without HRH (CLA-coated PTX-SPIONs) was included to distinctively discern the targeting ability imparted by the peptide. A significant decline in VEGF-A levels in HMEC-1 treated with CLA-coated PTX-SPIONs@HRH compared to untreated cells is indicative of a possible disruption in normal VEGF-A/VEGFR signaling. This agreed with the proposed notion that CLA-coated PTX-SPIONs@HRH could halt angiogenesis through selective binding of the nanosystem to VEGFRs, and could explain the relative good cellular uptake and anti-proliferative activity demonstrated by CLA-coated PTX-SPIONs@HRH (Figures 5 and 6). Fundamentally, growing cells continuously secrete VEGF-A which binds to VEGFR 2 for further growth and migration, as VEGFR 1 has minimal kinase activity.^44^ Because VEGF-A/VEGFR 2 downstream signaling regulates angiogenesis and stimulates endothelial proliferation, a decline observed in VEGF-A levels could be explained as limited cell proliferation due to inhibition of angiogenesis by VEGFR blockage. Herein, it is presumed that CLA-coated PTX-SPIONs@HRH bind VEGFR 2 and block the binding of VEGF-A, thus preventing further vascular development and epithelial proliferation.

Meanwhile, HMEC-1 treated with CLA-coated PTX-SPIONs exhibited comparatively increased levels of VEGF-A, indicative of continuous epithelial proliferation. This could hypothetically be the response of HMEC-1 in trying to overcome the exogenous agent by secreting more growth factors to stimulate further proliferation. Moreover, HMEC-1 are known to retain most of the primary angiogenic features and possess stability over time.^45^ Interestingly, the observation could suggest that even though CLA-coated PTX-SPIONs exhibit cancer anti-proliferative merits, their mechanism of action is different from that of CLA-coated PTX-SPIONs@HRH and does not involve angiogenesis inhibition.

### Antitumor Activity on Subcutaneous Lung Cancer Xenograft

**Figure 8:**
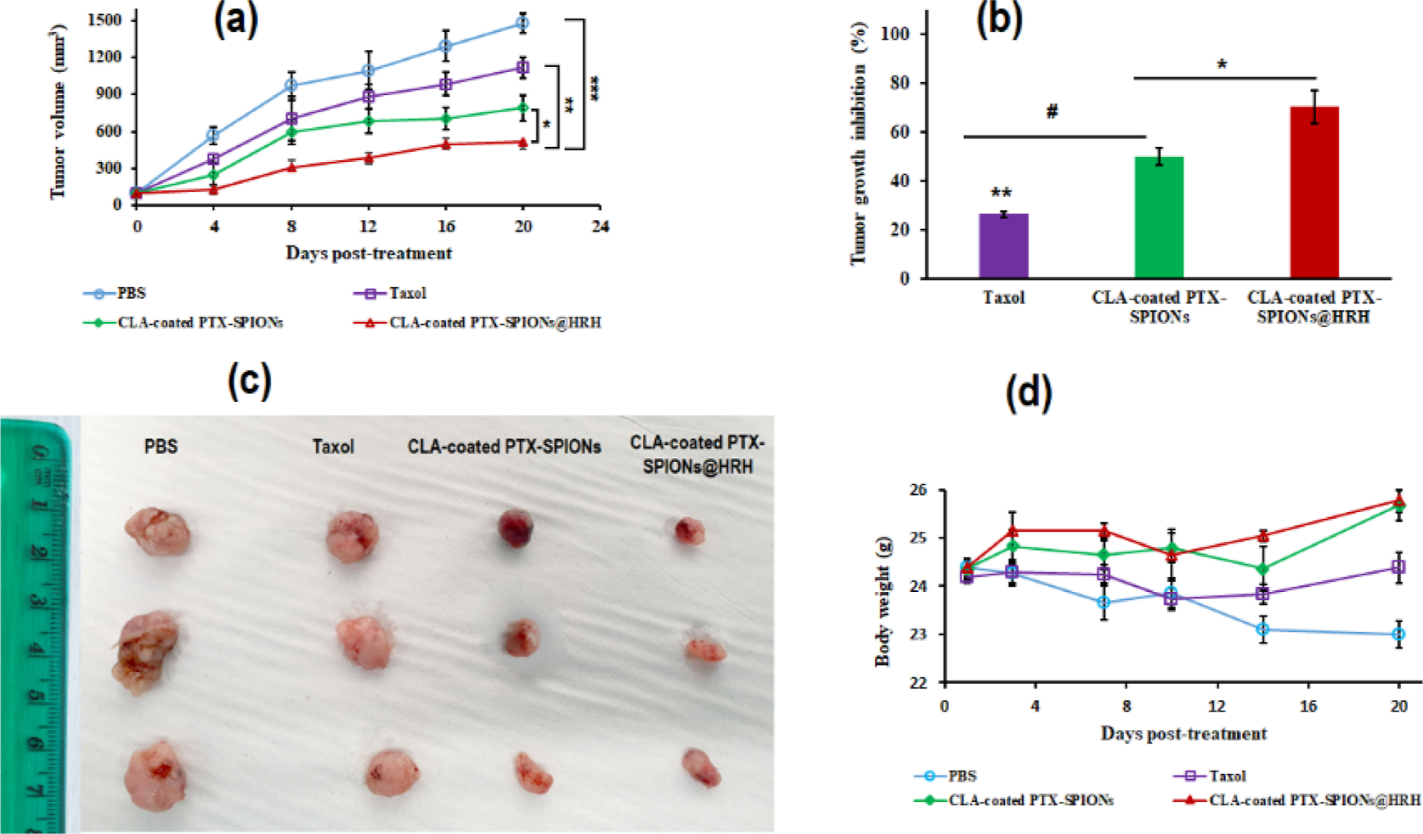
Representation of antitumor activity on an established SC lung tumor Xenograft model, showing (a) variation in tumor volumes post treatment, (b) % TGI determined at the end of the study, (c) images of excised tumors from mice, and (d) body mass of tumor-bearing mice over the duration of the study. Data depicted as mean ± SD, *n* = 3 (*p < 0.05, **p < 0.01, and ***p < 0.001, compared to CLA-coated PTX-SPIONs@HRH, and *#* denotes *p* < 0.05, compared to PTX).

Subcutaneous (SC) tumor Xenograft models are widely used in cancer research, and nude mice are the most commonly used rodents for the establishment of xenografts models owing to their compromised immune system.^18,46^ In the present study, a lung tumor xenograft model was successfully established and used to evaluate *in vivo* antitumor activity of the formulated CLA-coated PTX-SPIONs@HRH. Accordingly, a rapid tumor growth was recorded in the PBS (placebo) group, with tumors reaching an average volume of 1484.7 mm^3^ at day 20 (Figure 8a). Tumor volumes in the similar range have been reported for SC lung tumor xenografts owing to the robustness of A549 cancer cells.^47^ After 20 days of treatment with SC injections of 12 mg/kg CLA-coated PTX-SPIONs@HRH, CLA-coated PTX-SPIONs, and 1.2 mg/kg Taxol, a significant regression in tumor volume was recorded in mice treated with CLA-coated PTX-SPIONs@HRH compared to the PBS, Taxol, and CLA-coated-PTX-SPIONs groups.

A tumor growth inhibition rate (% TGI) of 76.6, 52.8, and 26.3% was found in tumor-bearing mice treated with CLA-coated PTX-SPIONs@HRH, CLA-coated PTX-SPIONs, and Taxol, respectively (Figure 8b). Such a distinction in tumor growth variations among the groups could be visually confirmed from excised tumors (Figure 8c). The ability of CLA-coated PTX-SPIONs@HRH to significantly suppress tumor progression could be greatly attributed to the antiangiogenic activity of HRH peptide, which is primarily involved in halting tumor angiogenesis and starving the tumor of nutrients and oxygen.^10^ This subsequently prevents tumors from growing uncontrollably, as seen with the PBS group. In essence, the impact of the peptide was apparent as the non-peptide-functionalized nanoformulation (CLA-coated PTX-SPIONs = 52.8% TGI) could not match the antitumor activity of CLA-coated PTX-SPIONs@HRH (76.6% TGI); however, it showed improved tumor regression compared to Taxol (26.3% TGI).

Interestingly, no significant loss was noted in the weight of mice treated with CLA-coated PTX-SPIONs@HRH, CLA-coated PTX-SPIONs, and Taxol, meanwhile mice that received only PBS showed a notable weight loss (>5%) due to the progression of cancer over the duration of the study (Figure 8d). A loss of weight in skeletal muscle and adipose tissue, known as cachexia, is fairly common in cancer and may also present as a side effect to chemotherapy.^18,48^ As such, the results obtained indicated the potential of CLA-coated PTX-SPIONs@HRH and CLA-coated PTX-SPIONs to also retard cachexia, and further suggest that the dosage of Taxol administered could be tolerated, as no other side effects were observed.

### Tissue Histology of Selected Organs and Tumor Tissue

**Figure 9:**
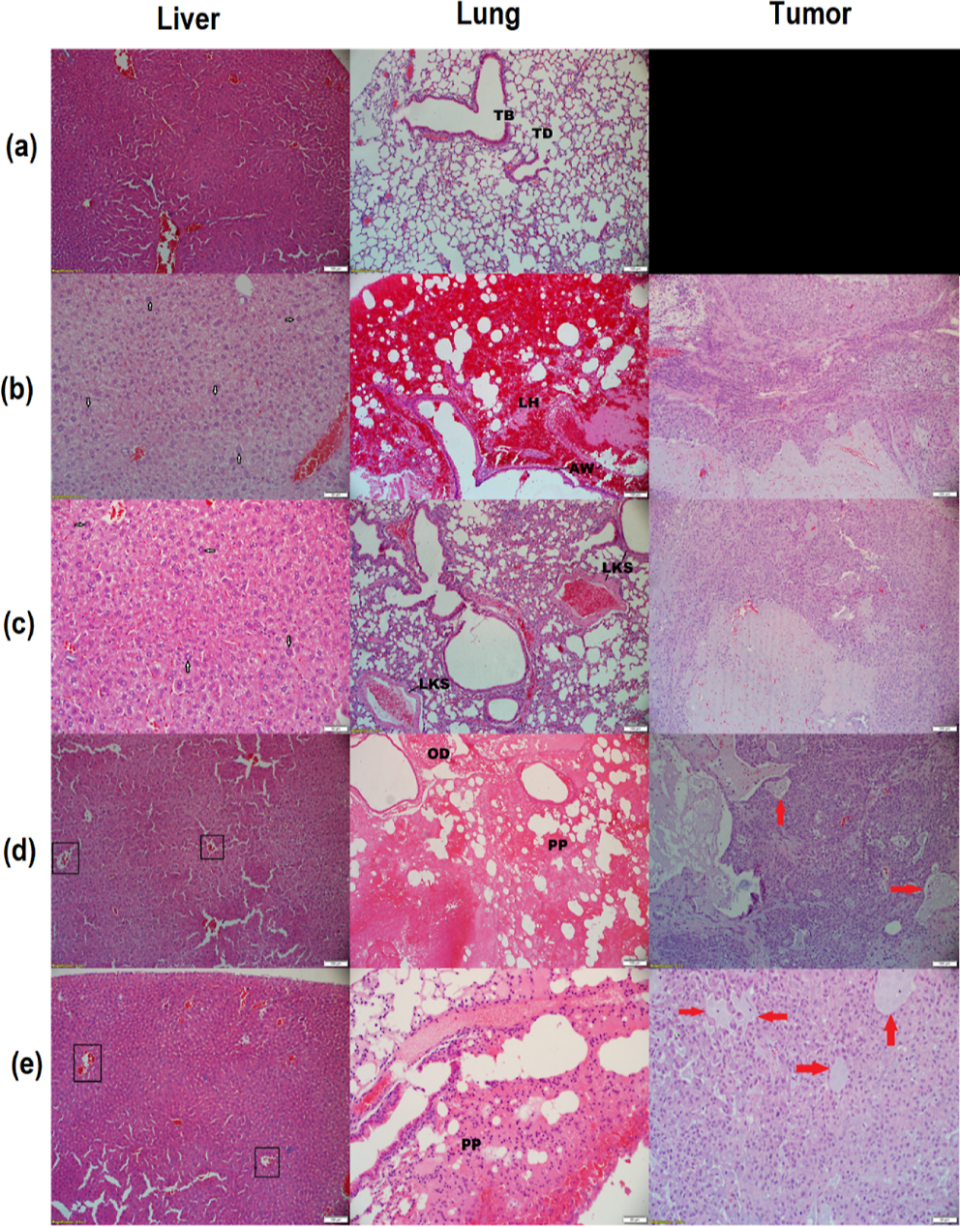
Histological analysis of liver, lung and tumor tissues from (a) normal healthy mice and tumor-bearing mice treated with (b) PBS only, (c) Taxol, (d) CLA-coated PTX-SPIONs, and (e) CLA-coated PTX-SPIONs@HRH. The images are generated from H&E stained tissue sections (∼5 μm) and captured at 100 μm, 6.3x magnification. Arrows with white fill show swollen hepatocytes identified on liver tissues from the mice that received PBS and Taxol, while red arrows show areas of degeneration of neoplastic cells, identified on tumors from CLA-coated PTX-SPIONs and CLA-coated PTX-SPIONs@HRH treated mice.

The histological analysis of liver, lung, and tumor tissues was conducted and is presented in Figure 9. Figure 9 shows the corresponding images (H&E staining) for (a) normal healthy mice, (b) PBS group, (c) Taxol group, (d) CLA-coated PTX-SPIONs group, and (e) CLA-coated PTX-SPIONs@HRH group. A normal histological appearance was observed in the liver tissues of healthy mice, as expected, whereas livers from tumor-bearing mice in the placebo (PBS) and Taxol groups exhibited prominent swollen hepatocytes (as depicted by arrows). Likewise, mildly swollen hepatocytes were identified in liver tissues from tumor-bearing mice in the CLA-coated PTX-SPION and CLA-coated PTX-SPIONs@HRH groups with occasional granular and finely vacuolated cytoplasm (squares). The observed swelling of hepatocytes and cytoplasmic vacuolization normally occur secondary to neoplastic-induced factors and are characteristic of the complicating effects of cancer.^49^

Lungs from normal healthy mice provided a good reference, showing a normal histological appearance with a characteristic terminal bronchiole (TB) leading to the alveolar duct (AD). Lungs from tumor-bearing mice in the PBS group exhibited a severe lobar hemorrhage (LH) and necrosis of the alveolar walls (AWs), while a mild leukostasis (LKS) was present in alveolar capillaries in the lungs of Taxol treated mice. Extensive hemorrhage could be seen throughout the disrupted pulmonary parenchyma (PP) in the lungs of tumor-bearing mice in the CLA-coated PTX-SPION and CLA-coated PTX-SPIONs@HRH groups, along with accumulation of edema (OD) fluid, yielding a fibrillar appearance in the former. These histological appearances could be associated with neoplastic-induced changes arising from the lung cancer that was induced in mice.

Notable histological variations were observed in tumors of the mice among the groups. Tumors from mice that received only PBS showed a multifocal inflammation surrounding muscle fibers along with edema. There were no signs of degeneration, with a record of 29 mitoses present in 10 high-power fields/2.37 mm^2^. Accordingly, the surrounding inflammation could be associated with the rapid growth of tumors, as inflammation is implicated in promoting stages of tumorigenesis.^50^ A cystic space was present in tumor mass of Taxol-treated mice, with few necrotic cells and loose red blood cells scattered throughout, and 20 mitoses could be spotted in 10 high-power fields. Tumors from mice treated with CLA-coated PTX-SPIONs and CLA-coated PTX-SPIONs@HRH all exhibited areas of degeneration of neoplastic cells (red arrows), more present in the CLA-coated PTX-SPIONs@HRH group (∼60% of the mass), with 8 and 2 mitoses present in 10 high-power fields/2.37 mm^2^, respectively. Essentially, the variation in the mitotic rate between the placebo and treatment groups validated the antimitotic effect of PTX, and a further decline recorded from tumors of mice treated with nanoformulations in this study supports our previous findings that CLA-coated PTX-SPIONs enhance the activity of PTX.^12^

### Plasma Pharmacokinetics of PTX

**Figure 10:**
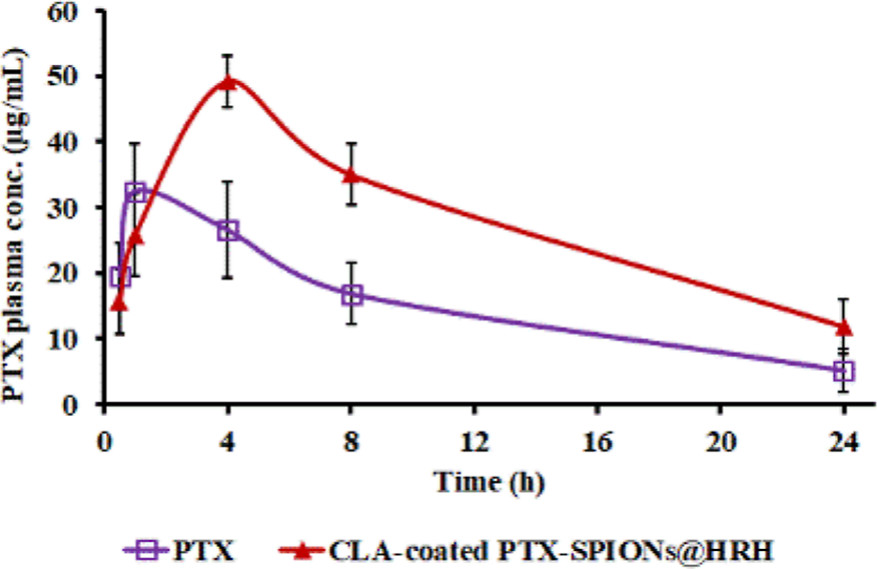
Plasma pharmacokinetic profile showing the concentration of PTX quantified from 0.5 to 24 h following SC injection of CLA-coated PTX-SPIONs@HRH and commercial PTX formulation. Data presented as mean ± SD, *n* = 3.

The concentration of PTX in plasma was determined following a single SC injection of equivalent PTX dosage of CLA-coated PTX-SPIONs@HRH and Taxol (commercial PTX formulation) in mice. The pharmacokinetics profile is presented in Figure 10 with corresponding pharmacokinetic parameters in Table 1. Notably, the plasma concentration of commercial PTX was relatively high on the onset, at 0.5 h (19.7 μg/mL) compared to that of CLA-coated PTX-SPIONs@HRH (15.6 μg/mL). Moreover, commercial PTX had a maximum plasma concentration (*C*_max_) of 32.5 μg/mL reached at 1 h of administration, meanwhile CLA-coated PTX-SPIONs@HRH reached *C*_max_ of 49.2 μg/mL at 4 h. The half-life (*T*_1/2_) of commercial PTX was estimated to be 9.0 h, which was substantially lower compared to *T*_1/2_ = 17.1 h of CLA-coated PTX-SPIONs@HRH. The area under the concentration–time curve (AUC_0–24_) was found to be 365.4 and 665.9 μg/mL·h for commercial PTX and CLA-coated PTX-SPIONs@HRH, respectively. Essentially, the pharmacokinetics (PK) data indicated that PTX from CLA-coated PTX-SPIONs@HRH is slowly absorbed and released into plasma following SC injection, which is consistent with the *in vitro* data which demonstrated a sustained release of PTX over time. A higher *C*_max_ and prolonged half-life could be attributed to the marked ability of the nanostructures to shield PTX from pre-systemic degradation and non-specific binding, as opposed to the commercial PTX formulation which results in reduced bioavailability and rapid elimination.

**Table 1:** Plasma PK Parameters of Commercial PTX Formulation (Taxol) and CLA-Coated PTX-SPIONs@HRH^a^

	*C*_max_ (μg/mL)	*T*_max_ (h)	*T*_1/2_ (h)	*K*_el_ (h^–1^)	AUC_0–24_ (μg/mL·h)
CLA-coated PTX-SPIONs@HRH	49.2 ± 5.2	4.0	17.1	0.03	665.9
Taxol^®^	32.5 ± 3.7	1.0	9.0	0.07	365.4

a *T*_max_, time taken to reach maximum concentration; *K*_el_, elimination rate constant.

Recently, the use of PTX in chemotherapy shows that the drug is riddled with various drawbacks, including a short half-life, hydroxylation by enzymes in the liver, rapid excretion, and nearly 90% of the drug binds to tissue-proteins.^3,51^ Moreover, the polyoxyethylated castor oil and anhydrous alcohol (Cremophor EL) used commercially to enhance the drug’s solubility present with detrimental side effects, such as peripheral neuropathy, hyperlipidemia, and hypersensitivity reaction.^51,52^ Interestingly, the formulated CLA-coated PTX-SPIONs@HRH show that enhancement in the bioavailability of PTX and the observed pharmacokinetic merits (i.e, enhanced half-life, circulation time, and reduced elimination rate) could be attributed mostly to the physicochemical properties of CLA-coated PTX-SPIONs@HRH. Most magnetic nanoparticles with sizes around ∼100 nm have been shown to evade the reticular endothelial system and withstand rapid systemic clearance, thus resulting in prolonged circulation.^2,53^

Although intravenous (IV) administration is still widely used to administer chemotherapeutics, emerging reports show that the route has some concerning limitations, including patient discomfort, likely occurrence of infections, and high cost.^54^ As such, SC administration was explored in the present study, as potential acceptable route of administration, circumventing IV short-falls. SC administration has been proven to be well-tolerated by patients due to less invasiveness and ease of application and offers controlled drug absorption with efficient bioavailability.^54,55^ In the present study, the application of SC injection was proven to be effective for administration of CLA-coated PTX-SPIONs@HRH, with indications of a controlled drug absorption into circulation with limited non-specific binding and pre-systemic degradation.

## Conclusions

In this study, we have successfully established an efficient fabrication methodology for surface-functionalization of SPIONs with an antiangiogenic HRH-peptide to formulate a targeted drug delivery nanosystem, capable of selectively binding to VEGFRs and block the binding of VEGF-A responsible for angiogenesis. The formulated CLA-coated PTX-SPIONs@HRH is safe-by-design and presents with high PTX loading capacity and a sustained, site-specific release. Moreover, the nanosystem exhibits a relatively good uptake and internalization by lung adenocarcinoma cells, with a marked *in vitro* anti-proliferative activity, emanating from a sustained release of PTX over time. Significant lung tumor targetability was attained in a lung tumor Xenograft model, with a recorded tumor growth inhibition rate of 76.6% in mice treated with CLA-coated PTX-SPIONs@HRH. The HRH peptide was shown to actively facilitate the direct targeting of VEGFRs expressed on lung tumors, and halted angiogenesis, resulting in the degeneration of neoplastic cells and subsequent tumor regression. In essence, CLA-coated PTX-SPIONs@HRH present a potentially viable targeted nanomedicine for NSCLC management, with specific angiogenesis targeting and direct drug delivery at tumor sites. Nonetheless, following this study, more extended preclinical investigations will be undertaken subsequently. The exploitation of the magnetic attributes of the nanosystem for imaging and enhanced tumor targeting using an external magnet is proposed for potential theragnostic application of CLA-coated PTX-SPIONs@HRH in the future.

## Methods

### Materials

Iron (II/III) chloride salts (FeCl_3_·6H_2_O and FeCl_2_·4H_2_O), CLA solution (10E, 12Z CLA in ethanol), sodium hydroxide (NaOH) pellets, Taxol, *meso*-2,3-Dimercaptosuccinic acid (DMSA), 4′,6-diamidino-2-phenylindole (DAPI), 1-ethyl-3(3-dimethylaminopropyl) carbodiimide (EDC), phosphate buffered saline (PBS) tablets, fluorescein 5(6)-isothiocyanate (FITC), *N*-hydroxysuccinimide (NHS), and dimethylformamide (DMF), were procured from Sigma-Aldrich Corp. (St. Louis, MO, USA). Pristine PTX and docetaxel (DTX) were sourced from DLD-Scientific (Durban, South Africa). The HRH peptide was purchased from Peptron Inc. (South Korea).

### Fabrication of CLA-Coated PTX-SPIONs

The fabrication of CLA-coated PTX-SPIONs was achieved through a three-step process, as previously described by Ngema *et al.*, 2022.^12^ Accordingly, SPIONs were fabricated by a co-precipitation method using FeCl_2_·4H_2_O and FeCl_3_·6H_2_O, 0.3 and 0.6 mol, respectively, under an inert atmosphere. Subsequently, SPIONs were coated with 10E, 12Z CLA, exploiting the carboxylic functionality of CLA, allowing chemisorption onto the SPION surface. PTX self-assembled loading was achieved, with PTX adsorbing onto CLA hydrophobic ends through spontaneous hydrophobic–hydrophobic interaction. A 10% (*w*/*w*) PTX was added to 100% (*w*/*w*) CLA-coated SPION suspension for loading. Adsorbed PTX and the loading capacity (% DLC) were quantified on a Cary 50 UV spectrophotometer (Varian Inc., Palo Alto, CA, USA), meanwhile, thermogravimetric analysis (TGA 4000, PerkinElmer Inc., Waltham, MA, USA) was employed to quantify the CLA content.^12^

### Carboxylic Acid Functionalization of CLA-Coated PTX-SPIONs

CLA-coated PTX-SPIONs were further functionalized with carboxylic acid (−COOH) groups to allow for HRH peptide conjugation, employing optimized methods by Martins *et al.*, 2021^56^ and Dilnawaz *et al.*, 2010,^20^ with modifications. Briefly, 1.6 M DMSA was prepared in DMF, and 20 mg CLA-coated PTX-SPIONs was added into 450 μL DMSA solution. The mixture was stirred continuously at 300 rpm (MSH10 Magnetic Stirrer, Labcon, Johannesburg, SA) for 24 h to allow for thorough functionalization, and thereafter centrifuged for 20 min at 13,500 rpm, 10 °C (TC-MiniSpin, TopScien, Ningbo, China), with three subsequent washes with ethanol. The functionalized sample was lyophilized and examined using FTIR spectroscopy (Spectrum-100, PerkinElmer Inc., Waltham, MA, USA) and double titration method to confirm functionalization and determine acid number, respectively.

### Free Carboxylic Acid Group Quantification

The amount of free −COOH on the surface of CLA-coated PTX-SPIONs, following DMSA coupling, was determined using a double titration method.^20,24^ This was done to ascertain the presence of −COOH groups and estimate the concentration of EDC/NHS needed for optimal HRH conjugation. A 15 mg lyophilized sample of CLA-coated PTX-SPIONs-DMSA was prepared in 5 mL 1 N NaOH to generate free COOH ends, for 30 min. The nanoparticles were washed three times by centrifugation at 13,500 rpm, 10 °C (TC-MiniSpin, TopScien, Ningbo, China) for 20 min, using deionized water, then lyophilized. The sample was collected into a flask containing 5 mL of deionized water and titrated to an endpoint with oxalic acid standardized solution of NaOH. The acid number (*A*) was computed using formula 1
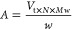
1where *V*_t_ is the required titration volume (mL), *N* is NaOH normality, *M*w denotes molecular weight of NaOH, and *w* is the sample weight (g).

### HRH Peptide Conjugation to CLA-Coated PTX-SPIONs

HRH was conjugated to COOH-functionalized CLA-coated PTX-SPIONs by employing the chemistry of EDC and NHS.^20,57,58^ A sample of functionalized CLA-coated PTX-SPIONs (10 mg) was prepared in 5 mL of 0.01 M PBS (pH 7.4). Solutions of NHS and EDC (15 mM each) were prepared, and 250 μL of each solution was added into the sample. The concentration of EDC/NHS was fixed at 1:0.5 molar ratio to free −COOH (as per calculated acid numbers). The mixture was then stirred at 200 rpm (MSH10 Magnetic Stirrer, Labcon, Johannesburg, SA) at ambient temperature for 4 h. A magnetic decantation was performed to remove the supernatant, followed by addition of 3 mL of PBS (0.01 M, pH 7.4) and 300 μL of HRH (1 mg/mL) into the pellet. This was acclimatized at ambient temperature (2 h) prior to overnight incubation at 4 °C. Subsequently, the sample was precipitated using a permanent magnet, washed with PBS (three times) to remove unbound peptide, and the supernatant was collected to determine % HRH conjugation. The sample was placed aside to dry at ambient temperature and the obtained CLA-coated PTX-SPIONs@HRH were further characterized. The % HRH conjugation was determined using a prepared standard plot of HRH concentrations (5–30 μg/mL) on a Cary 50 UV spectrophotometer (Varian Inc., Palo Alto, CA, USA) at 280 nm, using formula 2
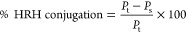
2where *P*_t_ is the total amount of peptide added and *P*_s_ is the amount of unbound peptide in the supernatant, in mg.

### Assessment of Chemical Functionality and Functional Transformations

Chemical composition of individual materials and functional transformations in the synthesized constituents of CLA-coated PTX-SPIONs@HRH were assessed using the FTIR spectroscopy (Spectrum-100, PerkinElmer Inc., Waltham, MA, USA). The analysis was conducted with the set conditions in place; 4000–550 cm^–1^, 120 psi, and 20 scans.^12^

### Analysis of Particle Hydrodynamic Size, Polydispersity Index, and Zeta Potential

The average hydrodynamic size, PDI, and zeta potential (surface charge) of CLA-coated PTX-SPIONs@HRH were ascertained using a dynamic Malvern NanoZS (Malvern Panalytical, Malvern, UK). Dynamic light scattering analyses were performed on a sample (10 μg/mL in distilled water) to confirm the particle size and PDI, while phase-analysis light scattering analyses confirmed the zeta potential. The analyses were conducted at 25 °C using a disposable cuvette and a DTS 1070 cuvette, for size/PDI and surface charge, respectively. The sample was first sonicated (Sonics Vibra Cell, Newtown, CT, USA) for 10 min prior to analyses.

### Assessment of the Overall Morphology

TEM as well as SEM was employed to investigate the overall morphology of CLA-coated PTX-SPIONs@HRH. An aliquot was withdrawn from the sample that was previously measured for hydrodynamic size and was allowed to dry on a specimen stub and coated twice with gold palladium (AuPd) for SEM analysis (ZEISS SEM, Carl Zeiss Microscopy Ltd., Cambridge, UK). Meanwhile, preparation for TEM analysis involved dipping a carbon-coated 200-mesh copper grid into a sample suspension and dried overnight before analysis (FEI Tecnai T12 120 kV, FEI Technologies Inc., Hillsboro, OR, USA).

### *In Vitro* PTX Release from CLA-Coated PTX-SPIONs@HRH

The release of PTX from CLA-coated PTX-SPIONs@HRH was evaluated at physiological pH (7.4) as well as tumor microenvironment-mimicking pH (6.8).^58^ A 3 mg sample of CLA-coated PTX-SPIONs@HRH was prepared in 2.5 mL of each corresponding 0.1 M buffer (pH 6.8 and 7.4) conditioned with 0.1% tween (*v*/*v*), in a dialysis membrane (SnakeSkin, 3500 MWCO). The membrane only permitted the release of PTX into the release buffer over 24 h in a 37 °C orbital shaker (YIHDER LM530, YIHDER Co., Lt., Taipei, Taiwan). The amount of PTX released at *t* = 1, 2, 4, 8, 12, 16, and 24 h was computed from the standard curve at 227 nm (Cary 50, Varian Inc., Palo Alto, CA, USA). Medium replenishment with equivalent sampling volume (2 mL) after each sampling point was carried out at all sampling points.

### Anti-proliferative Activity Assessment on Lung Adenocarcinoma

A lung adenocarcinoma (A549) cell line was employed to assess the viability of the cells treated with CLA-coated PTX-SPIONs@HRH, over 72 h. A standard 3-(4,5-dimethylthiazole-2-yl)-2,5-diphenyltetrazolium bromide (MTT) protocol was followed (MTT Cell Proliferation Kit L, Roche, Basel, Switzerland). Dulbecco’s Modified Eagle Medium (DMEM) with 1% *v*/*v* penicillin–streptomycin antibiotic and Fetal Bovine Serum (10% *v*/*v*, FBS) supplements was used to culture cells. The cells were incubated at 37 °C, 5% CO_2_ saturation, and humid environment until reaching a confluence of 90%. Cells were then seeded in a 96-well plate (2.5 × 10^4^ cells/ml), with each well containing 90 μL cell suspension, and incubated for 24 h (37 °C, 5% CO_2_) for optimal cellular adherence. Treatment concentrations of 25, 50, and 100 μg/mL of CLA-coated PTX-SPIONs@HRH and CLA-coated PTX-SPIONs were prepared in PBS, and experimental cells treated (*n* = 3) with 10 μL, while control cells were left untreated. The cells were subjected to incubation for 72 h (37 °C, 5% CO_2_, humidity) before the viability assay could be carried out. Accordingly, after 72 h, MTT solution (10 μL, 5 mg/mL) was added into wells and the plate incubated for 4 h as before. Thereafter, formed formazan crystals were dissolved with an acid-isopropanol solubilizing agent (100 μL) overnight under incubation. The plate was then analyzed using a multimodal microplate reader (Victor X3, PerkinElmer, Waltham, MA, USA) by measuring the absorbance at 570 nm. Plate wells with only the growth medium and solubilizing agent were sampled as a blank. The % cell viability (CV) was calculated from the absorbance readings (mean ± standard deviation) using formula 3
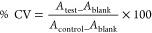
3where *A*_test_, *A*_control_, and *A*_blank_ are test absorbance, control absorbance, and blank absorbance, respectively, in nm (620 nm reference wavelength).

### Evaluation of Cellular Uptake and Internalization by Lung Adenocarcinoma Cells

FITC-labeled CLA-coated PTX-SPIONs@HRH were prepared using a modified encapsulation method described by Kumar and Srivastava (2018).^59^ Briefly, FITC (1 mg) was dissolved in 1 mL of dichloromethane and methanol (50:50), and the solution was transferred dropwise into a nanoformulation suspension (2.5 mg in 5 mL of distilled water). The sample was stirred at 300 rpm for 15 min (Magnetic Stirrer MSH10, Labcon, Johannesburg, SA) in the dark and at ambient temperature to evaporate the solvent. The sample was then centrifuged three times at 14,000 rpm, 10 °C for 20 min (TC-MiniSpin Centrifuge, TopScien, Ningbo, China) and subsequently washed (distilled water) to remove unbound FITC. The FITC-labeled nanoformulation was collected and stored away from light.

The model A549 cell line was cultured as described above and employed for the uptake and internalization study. Cells were seeded (5 × 10^4^ density) on presterilized cover slips in a 6-well plate, with each well containing 800 μL cell suspension, and incubated for 24 h at 37 °C and 5% CO_2_. After incubation, experimental cells were treated with 200 μL of FITC-labeled nanosystem (1 mg/mL in PBS) and further incubated for 24 h, while control cells were not treated. After 24 h, all wells were spiked with 500 μL of 4% paraformaldehyde (PFA) for 2 min to acclimatize the cells to the fixation solution. Thereafter, the media were decanted and cells were fixed with 1 mL of 4% PFA for 20 min. The cells were washed four times with 2 mL of 1X PBS and stained with DAPI (300 μL; 300 nM), followed by incubation for 5 min in the dark at ambient temperature. The stained cells on cover slips were further washed four times with 1X PBS, and then cover slips were mounted on glass slides with 80% *v*/*v* cooled glycerol and dried before microscopy analysis. A compound fluorescent microscope (Olympus IX51, Olympus Corporation, Tokyo, Japan) was used to view and capture images of the cells at 517 nm green fluorescence excitation (FITC) and 461 nm blue fluorescence excitation (DAPI) using the super 40X objective lens.

### Assessment of VEGFR Targeting Using Enzyme-Linked Immunosorbent Assay (ELISA)

A human dermal microvascular endothelial cell line (HMEC-1) (Separation Scientific SA Pty. Ltd, Johannesburg, South Africa) was employed for the evaluation of VEGFR targeting by CLA-coated PTX-SPIONs@HRH, using a Human VEGF-A ELISA Kit (Invitrogen, Thermo Fisher Scientific Corporation, Waltham, MA, USA). HMEC-1 cells were cultured in a special MCDB-131 medium augmented with 10 ng/mL human EGF recombinant protein, 1 μg/mL hydrocortisone, 10 nM l-glutamine, and 10% *v*/*v* FBS. The cells were incubated in a T-25 culture flask at 37 °C, 5% CO_2_ saturation, and humid environment. At 90% confluence, cells were seeded (5 × 10^4^ density) in two 6-well plates, with each well containing 800 μL cell suspension, and incubated for 24 h. Thereafter, cells were treated in triplicate with 200 μL of 50 μg/mL CLA-coated PTX-SPIONs@HRH and CLA-coated PTX-SPIONs for 24 h under incubation. The control cells were not treated. After 24 h of incubation, 1 mL of cell media supernatant was collected, placed on ice, and centrifuged at 13,000 rpm, 4 °C for 20 min (Eppendorf Centrifuge 5415R, Merck, Darmstadt, Germany). The supernatant was then used for VEGF-A ELISA analysis as per the manufacturer’s protocol, with the absorbance recorded at 450 nm using a multimodal microplate reader (Victor X3, PerkinElmer, Waltham, MA, USA).

### Development of a Subcutaneous Lung Tumor Xenograft Model

Athymic (MF1-nu/nu) female nude mice weighing 18–22 g (4–6 weeks old) were sourced and housed at the Wits Research Animal Facility, University of the Witwatersrand, with the study granted ethical clearance (clearance number: 2020/11/01/B) by the Animals Research Ethics Committee. The mice were kept in individual cages in a temperature and humidity-controlled room, with the provision of food and water *ad libitum* in a 12 h light/dark cycle. Mice were inoculated under anesthesia (2% isoflurane gas) with 3.9 × 10^5^ A549 cells *via* a subcutaneous (SC) injection of 50 μL of the cell suspension in PBS at the right flank. Tumors were visible 20 days post-inoculation and allowed to grow to a treatable volume of 90–100 mm^3^. The tumor volume (*V*) was computed from the measurements taken with a digital caliper, using formula 4(60)
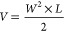
4where *W* is the tumor width and *L* is the length in mm (digital calliper).

### Evaluation of Antitumor Activity on Lung Cancer Xenografts

Tumor-bearing mice were subjected to treatment with formulated CLA-coated PTX-SPIONs@HRH, CLA-coated PTX-SPIONs, as well as commercial PTX formulation (Taxol), with the control group only receiving PBS. The mice were randomly divided into four groups (*n* = 3) and received 100 μL injection volume of the required dosage *via* SC injection (20 mm away from the tumor; right flank). The administered dosage of CLA-coated PTX-SPIONs@HRH and CLA-coated PTX-SPIONs was 12 mg/kg, while that of commercial PTX was 1.2 mg/kg (tolerated dosage equivalent to 10% dosage of our nanoformulation, corresponding to 10% DLC). Treatment was administered on day 0, 4, 8, and 12^47^ with tumor volumes routinely measured on each treatment day, before injection, and animal weights measured after every 2 days until day 20. The study was concluded on day 20 and the mice were humanely euthanized. Tumor growth inhibition (% TGI) was determined from the tumor volumes, using formula 5(61)
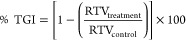
5where RTV_treatment_ is relative tumor volume of the treatment group (tumor volume on day 20/tumor volume on day 0) and RTV_control_ is the relative tumor volume of the control (PBS) group.

### Histological Analysis

Tumor, lung, and liver tissues were excised from tumor-bearing mice on the termination day (day 20). The excised tissues were kept in 10% neutral buffered formalin before being transferred into paraffin for sectioning (4–5 μm). Hematoxylin and Eosin (H&E) stain was applied for examination of histological variations in tissues, according to the IDEXX JB661428 protocol (IDEXX Laboratories Pty. Ltd, Johannesburg, South Africa).

### Pharmacokinetics Evaluation on Plasma

Mice were treated according to their assigned groups (*i.e.*, CLA-coated PTX-SPIONs@HRH or commercial PTX), with each group having 3 mice (*n* = 3), and euthanized over 24 h at *t* = 0.5, 1, 4, 8, and 24 h. Blood was collected at each time point *via* cardiac puncture into heparin-flushed microcentrifuge tubes and centrifuged at 10,000 rpm, 10 °C for 10 min (TC-MiniSpin Centrifuge, TopScien, Ningbo, China). The supernatant was collected and analyzed using high pressure liquid chromatography (Flexar LC UV/VIS, PerkinElmer Inc., Waltham, MA, USA) for the pharmacokinetics (PK) study. A validated rapid and sensitive high pressure liquid chromatography (HPLC) method was employed for the quantification of PTX from plasma using a liquid–liquid extraction technique.^62^ Briefly, plasma samples were thawed at ambient temperature followed by centrifugation at 13,000 rpm, 10 °C for 10 min (TC-MiniSpin, TopScien, Ningbo, China). The supernatant was isolated into microcentrifuge tubes, spiked with 100 μL of the docetaxel (DTX) internal standard, and vortexed for 1 min (BenchMixer Vortex 115 V, Thermo Fisher Scientific, Waltham, MA, USA). A 300 μL of 1.25% (*v*/*v*) ethanol in diethyl ether was added, and the sample mixture briefly vortexed and centrifuged again at 1300 rpm for 10 min. The resulting supernatant was collected into clean vials and air-dried. A 100 μL of mobile phase (60:40; acetonitrile/water) was added to reconstitute the residues and filtered before the analysis. A separation was carried out with a Waters C_18_ column (250 × 3.9 mm, 5 μm) (Waters Chromatography Ireland Ltd., Wexford, Ireland), with 20 μL injection volume and 1.9 mL/min flow rate, at a wavelength of 227 nm.

### Statistical Analysis

A two-tailed Student’s unpaired *t*-test was applied to statistically analyze the data on GraphPad prism 9 (GraphPad Software, Inc., San Diego, CA, USA), with *p* < 0.05 deemed statistically significant. Data are presented as mean (*n* = 3) ± standard deviation.

## Abbreviations

10E, 12Z CLA: *trans*-10,*cis*-12 conjugated linoleic acid
DAPI: 4′,6-diamidino-2-phenylindole
DMSA: *meso*-2,3-Dimercaptosuccinic acid
DTX: docetaxel
EDC: 1-ethyl-3(3-dimethylaminopropyl) carbodiimide
FITC: fluorescein 5(6)-isothiocyanate
HMEC-1: human dermal microvascular endothelial cell line
MTT: 3-(4,5-dimethylthiazole-2-yl)-2,5-diphenyl tetrazolium bromide
NHS: *N*-hydroxysuccinimide
NSCLC: non-small-cell lung carcinoma
PTX: paclitaxel
SPIONs: superparamagnetic iron oxide nanoparticles
VEGF: vascular endothelial growth factor
VEGFRs: vascular endothelial growth factor receptors
